# Medical Students at Philadelphia

**Published:** 1848-01

**Authors:** 


*Medical Students at Philadelphia.*—Philadelphia, as we learn from the Medical Examiner, still preserves its quota of medical students. The Examiner estimates the whole number in attendance at the various schools at about eleven hundred. The class at the University, we learn though a private source, embraces five hundred; so that the extension of the lecture term to five months, it would seem, has operated as an additional inducement for medical students to select that institution. We are not surprised at this. We believe Institutions which furnish the most advantages will be best patronized.

